# The Concussion Challenge Assessment: Development and reliability of a novel gross motor assessment tool for paediatric concussion

**DOI:** 10.3389/fspor.2022.1027339

**Published:** 2022-12-14

**Authors:** Michelle R. Tanel, Christopher Gupta, Katherine E. Wilson, James Murphy, F. Virginia Wright, Nick Reed

**Affiliations:** ^1^ Bloorview Research Institute, Holland Bloorview Kids Rehabilitation Hospital, Toronto, ON, Canada; ^2^ Department of Occupational Science and Occupational Therapy, Faculty of Medicine, University of Toronto, Toronto, ON, Canada; ^3^ Rehabilitation Sciences Institute, Faculty of Medicine, University of Toronto, Toronto, ON, Canada; ^4^ Department of Physical Therapy, Faculty of Medicine, University of Toronto, Toronto, ON, Canada

**Keywords:** concussion, youth, paediatric, gross motor performance, return to activity, development

## Abstract

**Aims:**

The aim of this study was to develop a gross motor performance clinical assessment tool, the Concussion Challenge Assessment (CCA), for paediatric concussion populations.

**Methods:**

An expert panel evaluated tasks from the Acquired Brain Injury Challenge Assessment to determine relevant tasks for a paediatric concussion population. These tasks were administered to a convenience sample of 854 healthy youth. An analysis of the response options for each task, considering task difficulty, was performed. The test–retest reliability of each task was considered to finalise the tool.

**Results:**

The Acquired Brain Injury Challenge Assessment was reduced to six tasks (three coordination, two speed and agility, and one strength) to create the CCA. Population-specific 4-point response options were generated, which, upon examination of task difficulty, were revised as 5-point response sets to better capture performance differences. The test–retest reliability results led to acceptance of all six: three performance tasks and three exertion tasks.

**Conclusion:**

This development of the CCA is an important step in creating a gross motor performance assessment tool that can assist in the determination of when youth are able to safely return to activity following a concussion.

## Introduction

An acquired brain injury (ABI) occurs after birth and can result from internal non-traumatic causes (i.e., brain tumour) or external traumatic causes (i.e., fall) (1). Traumatic brain injuries (TBI) are highly heterogeneous in their severity and can be classified as mild, moderate, or severe (2, 3). The incidence of traumatic brain injuries varies between 47 and 280 per 100,000, and approximately 80% of these are classified as mild (4). Concussions are defined as a mild traumatic brain injury (mTBI) induced by biomechanical forces (3). Concussions in the paediatric population are a growing concern as their prevalence has drastically risen in Canada, evidenced by a 4.4-fold increase in emergency department and physician office concussion-related visits across a 10-year period (5). It has been suggested that the developing brain may be more vulnerable to injury, which poses additional concern for concussions in the paediatric population (6). Poorer cognitive performance, prolonged recovery, less evidence of neural compensation, and increased risk of long-term functional deficits may occur following a concussion in youth (7–11).

The sequelae of symptoms following a concussion have been categorised as cognitive (e.g., difficulty concentrating), physical/somatic (e.g., headaches), or emotional/behavioural (e.g., lability) (3). Previous research has reported that cognitive as well as motor deficits may exist post-concussion (12–14). Researchers have questioned the exclusive use of neurocognitive testing in concussion evaluation and management, specifically in the paediatric population, due to considerable variability over time (3). Therefore, evaluating deficits in motor performance has recently come to the forefront as an additional way of tracking recovery. Motor deficits have been found to be more subtle in the concussion population compared to other brain injuries but may persist even 1 year post-injury (15). As skills associated with gross motor performance are critical in many sports (16), it is imperative that motor performance deficits are resolved prior to youth returning to sport. This has led to the recommendation of a multimodal approach to concussion evaluation and management, which includes both neurocognitive and motor components (3, 17). However, to our knowledge, there is no validated motor assessment tool specific to a paediatric concussion population. The unique aim of this study was to develop a gross motor assessment tool to address this critical measurement gap.

Despite the lack of validated measures of motor function for a paediatric concussion population, there are validated measures that can be used to evaluate advanced motor skill performance for youth with a brain injury including the Community Balance and Mobility Scale (18, 19), the High-Level Mobility Assessment Tool (20, 21), the Gross Motor Function Measure (22, 23), and the Bruininks–Oseretsky Test of Motor Proficiency (BOT) (22, 23). However, the items in these assessments do not sufficiently cover the motor skills needed to evaluate a high-functioning paediatric concussion population. Of these, the BOT has been used for children with mTBIs; it was concluded by the study authors that a more specific and sensitive measure with a more refined assessment of real-life activities is necessary for the mTBI population (23). Thus, the ceiling effect, lack of ecological validity, and original development of many of these measures for an adult population pointed to the need to create a gross motor assessment tool specific to the paediatric population that would be capable of assessing youth with higher level functional abilities.

The Acquired Brain Injury Challenge Assessment (ABI-CA) was developed for the paediatric brain injury population (24) and was modelled after the Gross Motor Function Measure as far as structure and underlying response format (24). The ABI-CA contains 20 items that examine four main components of gross motor performance: balance, coordination, strength, and speed and agility (25). Clinicians and children determined that these four components were essential to evaluate in the paediatric acquired brain injury population (24). The ABI-CA evaluates speed, distance, or number of repetitions in addition to the accuracy of the task (24). This unique quality of performance aspect of its scoring that is a departure from the Gross Motor Function Measure's extent of task completion focus, such that accuracy is required before performance speed is given credit on the ABI-CA (24). Although concussion falls under the broad umbrella of acquired brain injury, the ABI-CA was not created to meet the needs of the mTBI population. Specifically, the chosen tasks and cut points within its scoring were not created to capture subtle changes experienced by, for example, a high performance athlete. The developers of the ABI-CA speculated that it might have potential to be applied to high-functioning youth with acquired brain injury and those who have the potential to fully return to pre-injury motor performance (26).

Since the ABI-CA can assess more advanced gross motor performance, our team believed that an adapted version with more advanced scoring might be useful to assess gross motor performance in a concussion population. Therefore, the objectives of this study were to (1) refine the ABI-CA to a subset of tasks relevant to a paediatric concussion population and create the Concussion Challenge Assessment (CCA); (2) revise the item-specific response options and generate empirically based options specific to the youth athlete population; (3) evaluate the scoring of each item in the CCA; and (4) consider the test–retest reliability and finalise the CCA tool.

## Methods

Ethics approval for this research was obtained from the Holland Bloorview Research Ethics Board at the Holland Bloorview Kids Rehabilitation Hospital. All participants and their legal guardians provided signed informed consent prior to completing the study.

### Phase 1: Refinement of the ABI-CA to create the Concussion Challenge Assessment

The goal of this phase was to review and refine the ABI-CA so that it could be used for a paediatric concussion population. An expert panel was asked to determine a subset of tasks from the ABI-CA that could be used in concussion assessment and evaluation for a paediatric concussion population. This panel consisted of three clinicians (occupational therapist, physiotherapist, kinesiologist) who had 2–15 years of experience in testing advanced motor skills in youth with concussion, a neuropsychologist who had 10 years of experience in treating paediatric concussion, two researchers with 5–10 years of experience in researching assessment tools in paediatric concussion, and the senior scientist (paediatric physiotherapist with 15 years of experience in advanced gross motor skills testing children and youth with neuromotor conditions, including ABI) who led the development of the ABI-CA (FVW). In an in-person meeting, decisions to include or exclude tasks were made based on the gross motor performance components (strength, coordination, speed and agility, and balance) of each task and their representation of youth sports with higher risks for concussions (football, hockey, soccer, basketball) (5). The tasks were also assessed on whether they consisted of the skills for which youth might have difficulty post-concussion. Additionally, the feasibility and safety of administration was considered in determining the most appropriate tasks to include. The CCA would likely be part of a multimodal assessment; therefore, administration time constraints played a factor in the number of tasks that were to be included. An effort was made to include tasks such that collectively they would cover the various gross motor performance components (strength, coordination, speed and agility, and balance) evaluated in the ABI-CA. This panel also discussed the number of performance trials for each task that should be included in the assessment tool.

### Phase 2: Modification of item-specific response options

The goal of this phase was to develop empirically based response options specific to the youth athlete population. A convenience sample of 854 healthy youth athletes (ages 9–17 years) were recruited from the local community and sport organisations. As gross motor performance is vital to the participation of many sports (27) and youth athletes may perform differently than non-athletes on gross motor tasks, participants were required to play at least one sport recreationally or competitively in order to be included in this study. Participants were excluded from the study if they had a diagnosis of a developmental delay, a neurological condition, or could not read or write in English. Participants completed the multimodal baseline testing protocol outlined by Reed et al. including the six gross motor tasks from the CCA outlined in Table 1 (28). Eight members of the research team (kinesiologists and occupational therapists) who had been instructed by FVW on how the administer the CCA conducted the evaluations based on availability. Participants performed each of the CCA tasks following a demonstration by the trained assessor. Participants were instructed to complete each task as quickly as possible without committing any errors (be 100% accurate). The assessor recorded time/repetition/distance as well as number of errors committed and scored each item using the original ABI-CA response sets and cut points.

**Table 1:** Description of tasks from the ABI-CA included in the CCA.

Tasks from the ABI-CA	Description of Task from the ABI-CA^a^		
	Domain	Measure	Sample of Instructions given to Youth
Jumping Jacks	Coordination	Repetitions (i.e., number of jumping jacks), Errors (e.g., incorrect technique)	Jump from a standing position to make an “X” with your body and back again, without pausing, as fast and smooth as you can, until I say stop.
Pylon Obstacle Course	Speed and agility	Speed (i.e., time to complete), Errors (e.g., touches pylon)	Run in and out of the pylons to the end and back as fast as you can but at a speed that you feel safe. Do not touch the pylons.
Backwards Tandem Walking	Coordination, dynamic balance	Speed (i.e., time to complete), Errors (e.g., non-tandem step)	Walk backwards all the way along this line, keeping your toes touching your heel with each step. Walk as quickly and safely as possible. Make sure you stay on the line.
Modified Shuttle Run	Speed and agility, dynamic balance	Speed (i.e., time to complete), Errors (e.g., does not touch line)	Run to the end of this line, and one at a time pick up a beanbag and run back and put it in the basket. You must touch the starting line with your foot every time you come back.
Ins and Outs	Coordination	Speed (i.e., time to complete), Errors (e.g., breaks pattern)	Step out, step out, step in, step in, leading with the same foot every time. Do not step on the lines. Do this 10 times as quickly and safely as you can.
Standing Long Jump	Strength, dynamic balance	Distance (i.e., length of jump), Errors (e.g., falls)	From this line, jump forwards with both feet as far as you can. Land on both feet and freeze for 3 seconds. Don’t let your hands touch the floor.

ABI-CA, Acquired Brain Injury Challenge Assessment; CCA, Concussion Challenge Assessment.

^a^ Full description of set-up, cues, demonstrations, scoring criteria, and scoring notes are part of the required CCA training of the assessor and provided with the user access license to the measure.

**Figure 1:**
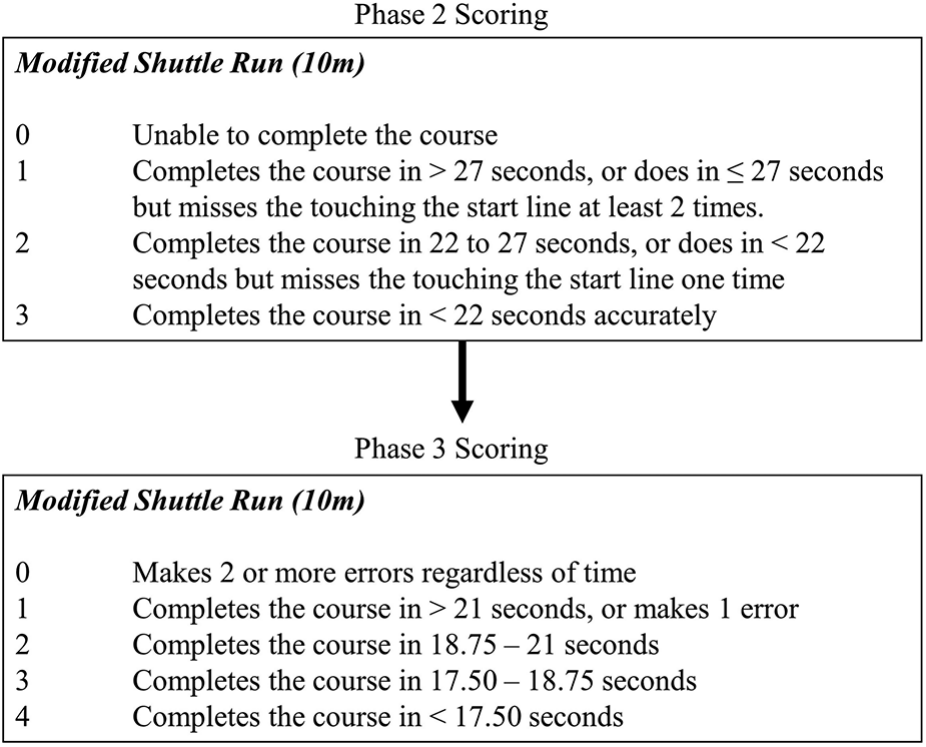
An example using the Modified Shuttle Run of the change in scoring from phase 2 to phase 3. Note: an error was missing the start line.

Item-specific cut points (time, repetitions, and distance) were generated for the CCA specific to the youth athlete population and were based on the process used for determining the ABI-CA cut points that would define a 4-point response scale (26). For example, a score of 4 is given to Standing Long Jump if the youth jumps 1.5 m with a clean landing and has no compensatory balance reactions as described in the scoring guidelines. The cut points (for time, repetitions, and distance) for each ABI-CA item had been empirically based and determined from testing the item set with 30 typically developing children aged 6–17 years (26). Cut points were derived from the mean and standard deviations (SD) for each item. More specifically, for tasks with a single cut point, the mean ± 1 SD was used. For tasks with two cut points, the mean ± 1 SD was used for the first cut point and the mean ± 2 SD was used for the second cut point (26). This same process was applied with the item data from our sample of 854 typically developing youth aged 9–18 years for the purpose of post-concussion evaluation. An example of scoring for tasks of the CCA with two cut points is provided in Figure 1. In accordance with the method used to determine the ABI-CA's cut points (26), only participants who completed the CCA tasks without error were included in developing the cut times, repetitions, or distances. This was done to ensure that speed/distance was not sacrificed at the expense of accuracy.

At this point, the consideration of accuracy of performance, expressed as performance errors made, was applied to the scoring system following the same approach that was taken with the ABI-CA (24). Specifically, scoring of errors was built into the response sets and incorporated compensatory movements and accuracy of movements as an added consideration to time, repetition, or distance results. Error scoring was included in the CCA to ensure that time, speed, or distance was not prioritised over accurate completion of the tasks.

### Phase 3: Evaluation of the scoring of items in the Concussion Challenge Assessment

The goal of this phase was to further develop the CCA. The investigative team conducted an in-depth review of the tasks in the CCA, evaluating each based on its level of difficulty. An analysis of the errors and distribution of the scores was used to evaluate the difficulty of each task. This first version of the CCA followed the same 4-point scoring system as the ABI-CA such that a score of “0” reflected inability to complete the task while a score of “3” reflected optimal performance. A mean score of at least 2.0 out of 3.0 with a SD of “1.0” was considered acceptable during the development of the ABI-CA tasks (26), and this criterion was initially applied to the CCA items when evaluating the scoring following the insertion of item-specific cut points derived from the youth athlete population.

### Phase 4: Test–retest reliability of the Concussion Challenge Assessment tool

The goal this phase was to consider the test–retest reliability of the CCA and to finalise the tool. An examination of the test–retest reliability for each task was conducted with 67 healthy youth athletes (9–18 years) recruited from the local community and sport organisations. The participants completed the multimodal baseline testing protocol outlined by Reed et al. at two different time points, but to be eligible for the post-test could not have sustained a concussion in the interim. The time between pre-test (time 1) and post-test (time 2) was different for each participant, varying from 2 weeks to 24 months, to replicate and assess conventional time variability between assessments in paediatric concussion. The assessors varied between pre-test and post-test based on availability between eight trained members of the research team. The assessor recorded time/repetition/distance as well as number of errors committed. Reliability of each task was analysed for the entire sample using intra-class correlation coefficients (ICCs) (29), with additional analyses completed separately for two clinically meaningful time intervals (0–12 and 12–24 months), age group (9–12 and 13–18 years), and sex (male and female). In accordance with previous literature, we considered a single ICC < 0.60 to denote unstable reliability, an ICC between 0.60 and 0.70 to reflect marginal reliability, and an ICC > 0.70 to indicate stable test–retest reliability (30–32). Upon review by the expert panel, a further modification of the CCA considered the option of creating cut points stratified by age and sex using scores from the sample described in step 2 as the cut point estimates in the manner described in phase 1.

## Results

### Phase 1: Refinement of the ABI-CA to create the Concussion Challenge Assessment

Following the discussion with the expert panel, the 23 item ABI-CA was reduced to 6 items to create the CCA. The six items chosen were Jumping Jacks, Backwards Tandem Walking, Ins and Outs, Pylon Obstacle Course, Modified Shuttle Run, and Standing Long Jump (Table 1). These represented three coordination tasks, two speed and agility tasks, and one strength task. Three of these tasks (Backward Tandem Walking, Modified Shuttle Run, and Standing Long Jump) also incorporated dynamic balance. All of these tasks included a speed, distance or repetition component, and an accuracy (error) component.

Through the discussion with the expert panel, it was also determined that one trial per task would be most appropriate to assess the performance in five of the six tasks rather than the two trials given with the ABI-CA. It was felt that the single attempt would better represent what the youth would do in the spontaneous context of sport and reduce the time constraint of the assessment. For the sixth task, Standing Long Jump, three trials were permitted where the maximum jump distance would be scored. Participants were encouraged to solidly land their first jump attempt and on subsequent attempts try to surpass that distance. This allowed participants to demonstrate maximum effort, knowing that at least one of their jumps was successful. Three trials for this task are also common in other physical testing protocols including the EUROFIT (33).

### Phase 2: Generation of item-specific response options

Item-specific cut points were established using the means and standard deviations of participants who completed the tasks without any errors. Once established, these cut points were applied to score all participants. Based on the performance of the 854 participants, timing cut points increased (became easier) for some tasks by up to 12 s and decreased (became harder) for some tasks by up to 5 s compared to the cuts determined with a sample of typically developing youth in the original ABI-CA. Distance for Standing Long Jump in addition to the number of repetitions for Jumping Jacks increased for each cut point following the establishment of new cut points.

### Phase 3: Evaluation of the scoring of items in the Concussion Challenge Assessment

As all participants were healthy youth athletes, all completed the six tasks and many participants had near optimal performance. Five tasks (Jumping Jacks, Pylon Obstacle Course, Modified Shuttle Run, Ins and Outs, and Standing Long Hump) had mean scores greater than 2.0 out of 3.0 and negatively skewed distributions with many of the participants obtaining a perfect score (i.e., evidence of a ceiling effect). Backwards Tandem Walking had a mean score less than 2.0 indicating that the current scoring system might be too difficult. Thus, further alteration of the scoring appeared necessary for all tasks.

After a discussion with the expert panel, it was determined that a 5-point response scale in addition to revising the error scoring would be appropriate in order to normalise the distribution. The development of the 5-point response scales (0–4) for each of the six items involved using quartiles to determine the cut points. This is similar to the method used by Glazebrook and Wright in the development of the Challenge Assessment, an advanced motor skills assessment used for children with cerebral palsy (34) that was developed in parallel with the ABI. An examination of the number of participants in the CCA test sample committing errors informed the subsequent revision of error scoring. For Backwards Tandem Walking, the task where committing errors was most common, reanalysis of the data using the quartiles meant that the maximum score attainable with just one error committed was adjusted to be a 3 out of 4. For Ins and Outs, where committing errors occurred less frequently, a maximum score of 2 out of 4 could be assigned with one error. For Modified Shuttle Run, where errors were less common than Ins and Outs, a maximum score of 1 out of 4 would be assigned with one error. For the remaining three tasks (Jumping Jacks, Pylon Obstacle Course, and Standing Long Jump), less than 5% of participants committed an error and, therefore, an error automatically resulted in a revised score of 0. These response option modifications for the error ratings for each task are explained in Table 2.

**Table 2:** Error scoring modification by task in the Concussion Challenge Assessment.

Task	Observation	Modifications to response options (0 to 4 scale)
Jumping Jacks	Errors very rarely committed (1.8% of participants).	Score of 0 given if one or more errors committed, regardless of number of jumping jacks completed. Scores of 1–4 reflect number of jumping jacks completed.
Pylon Obstacle Course	Few participants (2.4%) committed an error.	Score of 0 given if one or more errors committed, regardless of time. Scores of 1–4 reflect differences in time to complete.
Backwards Tandem Walking	Task where greatest number of participants committed errors (55.3%). Scores of 1 and 2 were most common.	Score of 0 given if five or more errors are committed, regardless of time. Scores of 1–3 reflect combination of time and number of errors committed. Score of 4 reflects no errors committed and considers time required to complete.
Modified Shuttle Run	Few participants (11.8%) committed errors.	Score of 0 given if two or more errors are committed regardless of time. Score of 1 reflects combination of time and/or one error committed. Scores of 2–4 reflect when no errors committed and based on time to complete.
Ins and Outs	Notable number of participants (25.5%) committed errors.	Score of 0 given if three or more errors committed regardless of time. Scores of 1 and 3 reflect time to complete task and when one or two errors committed. Scores of 3 and 4 reflect when no errors committed and based on time to complete.
Standing Long Jump	The majority of participants able to land at least one of their three jumps. Only 3.6% if participants unable to land at least one jump.	Score of 0 given if unable to perform a two-foot take off and/or landing. Scores of 1–4 reflect distance of successful jump.

### Phase 4: Reliability and finalisation the Concussion Challenge Assessment tool

Table 3 shows the ICCs for the entire sample and for each time interval, in which Backwards Tandem Walk, Ins and Outs, and Standing Long Jump achieved the target ICC >0.70 when the second assessment was within a <1- to 12-month time interval. Based on these results, these three tasks were considered to be suitable for inclusion in the CCA in its planned use as an outcome measure (follow-up assessment post-concussion). Following a discussion with the expert panel, it was determined that the three tasks that did not meet the test–retest reliability target (i.e., Jumping Jacks, Pylon Obstacle Course, and Modified Shuttle Run) could still yield beneficial clinical information when used as single point in time exertion tasks. Each of these tasks involve aerobic exercise that requires full body movement. None of the six tasks were considered to be reliable between the 12- and 24-month time intervals.

**Table 3:** Test–retest reliability results showing the mean standard deviation, and single ICCs for each task, and grouped by time interval, age, and sex.

		Mean (SD)		Single ICC	ICC (95% CI)	
Variable	Group	Time 1	Time 2		Lower	Upper
Jumping Jacks (#)	Time interval (months)					
	0–12 (*n* = 52)	22.97 (2.67)	23.44 (2.60)	0.56	0.34	0.72
	12–24 (*n* = 14)	24.14 (2.41)	24.14 (2.03)	0.24	−0.31	0.67
	Age (years)					
	9–12 (*n* = 26)	22.69 (2.46)	24.27 (2.03)	0.39	0.01	0.67
	13–18 (*n* = 26)	22.04 (2.88)	22.62 (2.87)	0.66^a^	0.37	0.83
	Sex					
	Male (*n* = 24)	21.79 (2.78)	23.21 (2.65)	0.70^b^	0.41	0.86
	Female (*n* = 28)	22.86 (2.52)	23.64 (2.59)	0.42	0.07	0.69
Pylon Obstacle (s)	Time interval (months)					
	0–12 (*n* = 50)	6.82 (0.75)	6.71 (0.71)	0.38	0.12	0.59
	12–24 (*n* = 14)	6.77 (0.69)	7.12 (0.61)	−0.34	−0.73	0.21
	Age (years)					
	9–12 (n = 26)	6.95 (0.84)	6.61 (0.52)	0.35	−0.04	0.65
	13–18 (*n* = 24)	6.67 (0.64)	6.82 (0.86)	0.49	0.11	0.74
	Sex					
	Male (*n* = 23)	6.51 (0.51)	6.31 (0.50)	0.45	0.05	0.72
	Female (*n* = 27)	7.08 (0.83)	7.05 (0.69)	0.14	−0.25	0.49
Backwards Tandem Walking (s)	Time interval (months)					
	0–12 (*n* = 17)	19.06 (5.91)	19.56 (6.85)	0.83^b^	0.60	0.94
	12–24 (*n* = 7)	19.38 (5.81)	19.58 (5.86)	0.63^a^	−0.14	0.92
	Age (years)					
	9–12 (*n* = 4)	17.72 (1.77)	18.44 (3.24)	−0.74	−0.98	0.40
	13–18 (*n* = 13)	19.47 (6.71)	19.90 (7.71)	0.88^b^	0.66	0.96
	Sex					
	Male (*n* = 5)	16.83 (5.34)	16.56 (5.61)	0.90^b^	0.34	0.99
	Female (*n* = 12)	19.99 (6.10)	20.80 (7.15)	0.81^b^	0.45	0.94
Shuttle Run (s)	Time interval (months)					
	0–12 (*n* = 39)	18.67 (1.55)	18.67 (1.84)	0.48	0.19	0.69
	12+ (*n* = 14)	19.79 (1.61)	19.00 (1.21)	0.46	−0.07	0.79
	Age (years)					
	9–12 (*n* = 19)	18.64 (1.52)	18.68 (1.36)	0.55	0.14	0.80
	13–18 (*n* = 20)	18.70 (1.62)	18.66 (2.25)	0.44	0.01	0.73
	Sex					
	Male (*n* = 18)	18.35 (1.76)	17.52 (1.34)	0.53	0.10	0.79
	Female (*n* = 21)	18.94 (1.33)	19.65 (1.65)	0.41	−0.02	0.71
Ins and Outs (s)	Time interval (months)			** **		
	0–12 (*n* = 39)	6.82 (1.55)	6.73 (1.42)	0.73^b^	0.54	0.85
	12+ (*n* = 8)	7.66 (1.12)	7.49 (0.80)	−0.40	−0.84	0.36
	Age (years)					
	9–12 (*n* = 18)	7.03 (1.67)	6.77 (1.57)	0.80^b^	0.54	0.92
	13–18 (*n* = 21)	6.63 (1.45)	6.71 (1.32)	0.65^a^	0.31	0.84
	Sex					
	Male (*n* = 15)	6.38 (1.50)	6.38 (1.70)	0.78^b^	0.47	0.92
	Female (*n* = 24)	7.09 (1.54)	6.96 (1.20)	0.66^a^	0.36	0.84
Standing Long Jump (m)	Time interval (months)			** **		
	0–12 (*n* = 50)	79.57 (7.25)	80.38 (8.24)	0.81^b^	0.68	0.89
	12+ (*n* = 14)	74.25 (9.93)	78.18 (7.76)	0.56	0.06	0.83
	Age (years)					
	9–12 (*n* = 25)	79.80 (7.63)	79.86 (7.85)	0.83^b^	0.65	0.92
	13–18 (*n* = 25)	80.34 (6.92)	80.90 (8.75)	0.80^b^	0.56	0.90
	Sex			** **		
	Male (*n* = 23)	83.89 (6.45)	85.22 (7.34)	0.76^b^	0.51	0.89
	Female (*n* = 27)	75.89 (5.76)	76.26 (6.61)	0.69^a^	0.42	0.84

ICC, intra-class correlation coefficient.

^a^ Reflect marginal reliability (ICC between 0.60 and 0.70).

^b^ Stable reliability (ICC > 0.70).

Following the planned discussion with the expert panel and review of the literature, it was determined that age- and sex-specific cut points should be generated for each task of the CCA, but the error rating decisions should remain constant across all participants. Separate cut points, using the same quartile method mentioned in phase 3, were subsequently generated for male and female children (aged 9–12 years) and male and female adolescents (aged 13–18 years) and then evaluated within test–retest analyses (Table 3). For the three reliable tasks, majority of subgroups had stable reliability (ICC > 0.70) and select subgroups (Ins and Outs: adolescents, females; Long Jump: females) had marginal reliability (ICC between 0.60 and 0.70), with only one subgroup (children) on the Backwards Tandem Walking task that did not meet target reliability.

## Discussion

A three-item CCA was developed with four versions to support age- and sex-specific scoring (cut points) with the added set of three items to give the option to also assess exertion. The CCA summary score that would be used for follow-up of a child is that of the three items combined, while the exertion items are standalone scores that provide a picture of this critical element of participation in sport.

### Phase 1: Refinement of the ABI-CA to create the Concussion Challenge Assessment

During this phase, it was important to include tasks consisting of various components of gross motor performance. The six tasks chosen reflect speed and agility, coordination, strength, and balance components of gross motor performance. It was critical to include a wide variety of gross motor performance components as these skills are involved in the participation of many sports (28), and thus assessing the performance areas required for sport participation are important in determining if an individual is able to return to play. Additionally, many of these tasks include the integration of motor and cognitive components. For example, the Modified Shuttle Run includes running and concurrently remembering to place a bean bag in a basket and touching the start line with one foot on every return trip. Multi-domain tasks may provide important information when assessing return to play following a concussion (35) and may be able to detect deficits not apparent when assessing motor or cognitive abilities independently. Therefore, a gross motor assessment tool including cognitive–motor integration within a multimodal assessment may be able to provide further information into an athlete's safe return to physical activity and sport/game play.

### Phase 2: Generation of item-specific response options

The goal of this phase was to establish item-specific cut points for each task specific to the youth athlete population. With the cut points established, the next step of evaluating the appropriateness of the scoring could be completed.

### Phase 3: Evaluation of the scoring of items in the Concussion Challenge Assessment

**Figure 2:**
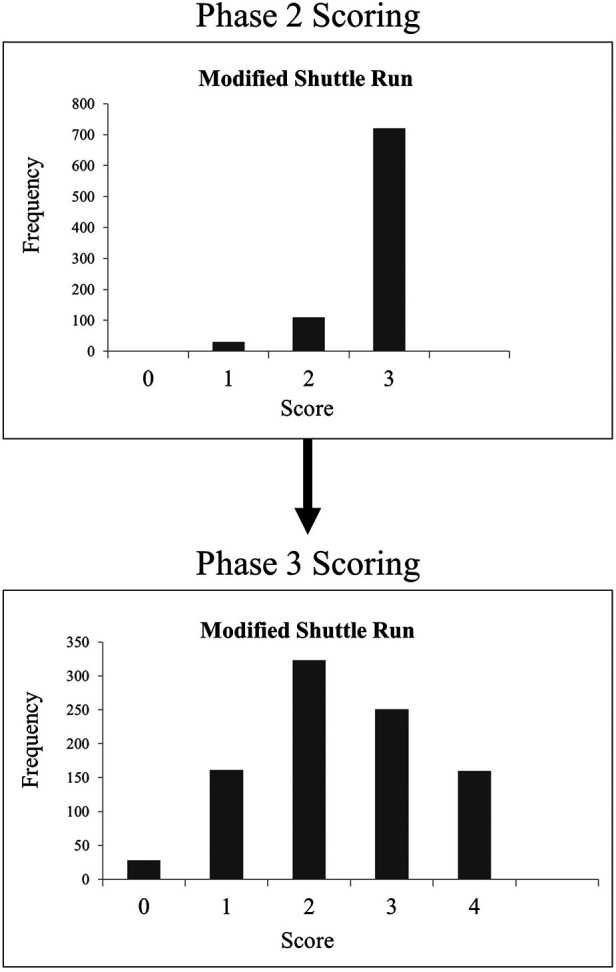
An example using Modified Shuttle Run of the change in distribution of CCA scores from phase 2 to phase 3. CCA, Concussion Challenge Assessment.

The importance of taking an alternative approach to scale development and evaluating first round data for a new scale and responding to scoring issues by using evidence to further adapt the scale was clearly demonstrated in this aspect of our work. The analysis of the distribution of scores following the implementation of item-specific cut points and the original error scoring revealed that five tasks (Jumping Jacks, Pylon Obstacle Course, Modified Shuttle Run, Ins and Outs, and Standing Long Jump) appeared to be exhibiting a ceiling effect. These tasks displayed negatively skewed distributions with many individuals attaining the highest score possible (36). This is problematic since many youth athletes can easily obtain a perfect score while healthy, and thus it is possible that post-concussion, youth may still achieve a perfect score while demonstrating a decrease in gross motor performance. In order to facilitate this measure's ability to be sensitive to change, especially in demonstrating decreases in performance, the 4-point scoring system was changed to a 5-point scoring system with more stringent top-level (score of 4) cut points as well as a revision of the error scoring to better capture the individual difficulty of the items. This added response level at the upper end of the scale allowed the distribution of scores to shift from a negative skew to a normal distribution (Figure 2). This in turn decreased the percentage of youth able to achieve a perfect score, minimising the ceiling effect previously observed.

### Phase 4: Reliability and finalisation of the Concussion Challenge Assessment tool

The development of a clinical assessment tool capable of assessing motor performance over a follow-up time period in a paediatric concussion population is valuable as part of the assessment of recovery following a concussion. The finalised version of the CCA has the potential to contribute to this understanding. Speed and agility, coordination, strength, and balance are important in sport participation (16); therefore, determining if deficits are detected during advanced motor skill performance in these domains should be helpful in return to sport and activity decision making. Its three selected tasks, Backwards Tandem Walking, Ins and Outs, and Standing Long Jump, can assist in evaluating if deficits in these areas of gross motor performance are detected following a concussion as they can be compared to baseline values within a 1-year time interval or norm-referenced values, once established. Exercise tolerance is also an important component to assess during concussion evaluation as it can prevent the premature resumption of full sport participation (37). Jumping Jacks, Pylon Obstacle Course, and Modified Shuttle Run can serve as single assessment exertion tasks as they can challenge youth to perform aerobic movements with speed. They do not appear to have the longer term measurement ability, however, for inclusion in the outcome item set of the CCA. The final measure is feasible and can assist clinicians in detecting risk for injury and guide their return to activity decision making. However, additional psychometric testing is needed prior to clinical use to determine clinically meaningful scores in the concussion population.

The decision to make cut points in the CCA specific to child and adolescent groups and males and females was based on a review of the literature and discussion with the expert panel. Previous research has reported that as youth age, muscle mass, and body proportions increase, leading to improved performance on tasks involving strength, agility, coordination, and power (38, 39). Additionally, evaluating children (aged 5–12 years) differently from adolescents (aged 13–17 years) has been recommended in concussions protocols (8). Sex has been shown to influence motor performance as well (40). Other motor assessment tools, including the EUROFIT and the BOT, also consider age and sex in the interpretation of results (24, 41). The literature supporting different scoring for different ages and sexes as well as other assessment tools providing scoring specific to different ages and sexes lead to the creation of four assessment forms: female children (aged 9–12 years), male children (aged 9–12 years), female adolescents (aged 13–17 years), and male adolescents (aged 13–17 years).

### Limitations

Some limitations in this study should be acknowledged. The members in the expert panel involved in the decision making were all from one facility, leading to a possible bias; however, all members had numerous years of experience in several areas including clinical concussion evaluation, assessment development, and paediatric research. Because of the large sample size used to generate the item-specific cut points, multiple assessors conducted the CCA. The ABI-CA has been shown to have excellent inter-rater reliability (25) and to further minimise the risk, all testers underwent the same training prior to administering the assessments. Further research is needed now to investigate the inter- and intra-rater reliability of the CCA with youth who have had a concussion to determine if the CCA can be scored reliably in that context. It will also be important to further explore the use of the CCA as a pre-season assessment to set an individualised benchmark that can be used if the child subsequently sustains a concussion to guide follow-up testing and return to activity. This may entail exploring the estimated CCA administration time in healthy and concussed populations to provide a point of reference for researchers and clinicians considering its use, and conduct multicentre studies that include large samples of youth participants with concussion to further validate and determine responsiveness to change as well as user acceptability.

## Conclusion

The use of a clinical assessment tool capable of evaluating gross motor performance following a concussion can provide valuable information to help determine when an individual is able to safely return to play/activity. This in turn can help reduce the risk of returning to play/activity prematurely and, therefore, reduce the risk of further injury or prolonged recovery. The development of the first version of the CCA is an important step in creating a clinical assessment tool for gross motor skills that can be used within a paediatric concussion multimodal assessment. With further research utilising a case control design, for example, the tool may be capable of providing gross motor performance information for clinicians that can support their decisions in determining a youth athlete's concussion recovery and readiness for return to play/activity. This study demonstrates that these gross motor tasks are feasible, can be reliable over time, and are practical options to embed in paediatric concussion assessment tools.

## Data Availability

The original contributions presented in the study are included in the article, further inquiries can be directed to the corresponding author.
